# Characterization of the gene expression profile response to drought stress in *Haloxylon* using PacBio single-molecule real-time and Illumina sequencing

**DOI:** 10.3389/fpls.2022.981029

**Published:** 2022-08-16

**Authors:** Fang Yang, Guanghui Lv

**Affiliations:** ^1^School of Ecology and Environment, Xinjiang University, Ürümqi, China; ^2^Key Laboratory of Oasis Ecology, Ministry of Education, Ürümqi, China; ^3^Xinjiang Jinghe Observation and Research Station of Temperate Desert Ecosystem, Ministry of Education, Ürümqi, China

**Keywords:** drought stress, *Haloxylon ammodendron*, *Haloxylon persicum*, RNA sequencing, single-molecule real-time sequencing

## Abstract

Haloxylon ammodendron and *Haloxylon persicum* are important drought-tolerant plants in northwest China. The whole-genome sequencing of *H. ammodendron* and *H. persicum* grown in their natural environment is incomplete, and their transcriptional regulatory network in response to drought environment remains unclear. To reveal the transcriptional responses of *H. ammodendron* and *H. persicum* to an arid environment, we performed single-molecule real-time (SMRT) and Illumina RNA sequencing. In total, 20,246,576 and 908,053 subreads and 435,938 and 210,334 circular consensus sequencing (CCS) reads were identified by SMRT sequencing of *H. ammodendron* and *H. persicum*, and 15,238 and 10,135 unigenes, respectively, were successfully obtained. In addition, 9,794 and 7,330 simple sequence repeats (SSRs) and 838 and 71 long non-coding RNAs were identified. In an arid environment, the growth of *H. ammodendron* was restricted; plant height decreased significantly; basal and branch diameters became thinner and hydrogen peroxide (H_2_O_2_) content and peroxidase (POD) activity were increased. Under dry and wet conditions, 11,803 and 15,217 differentially expressed genes (DEGs) were identified in *H. ammodendron* and *H. persicum*, respectively. There were 319 and 415 DEGs in the signal transduction pathways related to drought stress signal perception and transmission, including the Ca^2+^ signal pathway, the ABA signal pathway, and the MAPK signal cascade. In addition, 217 transcription factors (TFs) and 398 TFs of *H. ammodendron* and *H. persicum* were differentially expressed, including FAR1, MYB, and AP2/ERF. Bioinformatic analysis showed that under drought stress, the expression patterns of genes related to active oxygen [reactive oxygen species (ROS)] scavenging, functional proteins, lignin biosynthesis, and glucose metabolism pathways were altered. Thisis the first full-length transcriptome report concerning the responses of *H. ammodendron* and *H. persicum* to drought stress. The results provide a foundation for further study of the adaptation to drought stress. The full-length transcriptome can be used in genetic engineering research.

## Introduction

Drought is one of the main environmental factors that restrict the survival, secondary metabolism, and productivity of plant species (Li et al., 2021). Under drought stress, plants undergo several changes, which eventually lead to the reduction in gas exchange and photosynthesis and a decrease in cell division and cell expansion due to reduced enzyme activities and energy deficiency (Thayale Purayil et al., 2020). To adapt to these conditions, plants employ a series of morphological, physiological, biochemical and molecular mechanisms, such as growth restriction (Gupta et al., 2020), reactive oxygen species (ROS) scavenging (Zhang et al., 2022) and transcriptional activation (Zhu, 2016). Functional proteins and regulatory proteins are two major categories of stress-inducible proteins that play an important role in plant adaptation to stress (Thayale Purayil et al., 2020). Regulatory proteins are involved in the regulation of downstream genes in stress response pathways. This includes different types of protein kinases and transcription factors (TFs) (Golldack et al., 2014).

In plants, abscisic acid-mediated signaling plays a central role in plant responses to stress (Kumar et al., 2019). Under drought stress, increased levels of abscisic acid (ABA) lead to binding to PYR/PYL, which changes the conformation of PYR/PYL protein, allowing PYR/PYL to interact with the negative regulator type 2 protein C phosphatase (PP2C) to form a temporary complex (ABA-PYR/PYL-PP2C), which can inhibit the activity of PP2C. The low expression of PP2C will slow down the inhibition of SNF1-related kinases (SnRKs), and the expression of SnRKs will induce stomatal closure and activate downstream drought stress related genes and adapt plants to water-deficient environments (Sah et al., 2016; Zhu, 2016). Under drought conditions, the regulation of drought-responsive genes is regulated by various TFs, such as the bZIP family, the AP2/ERF superfamily, and the NAC family. These TFs play important roles in plant defense through ABA-dependent and ABA-independent stress tolerance mechanisms (Yu et al., 2019). The mitogen-activated protein kinase (MAPK) cascade is one of the major abiotic stress response pathways involved in transduction of external stimuli to the nucleus to appropriately tune the cell’s response under stress (Zhang et al., 2022). The role of MAPK signaling cascades in abiotic stresses such as drought, salt stress, high temperature and low temperature stress has been elucidated in different plants (Bigeard and Hirt, 2018). Under drought conditions, the phosphorylation of target genes is regulated by MAPK, which controls the activities of different structural proteins and various TFs involved in abiotic stress tolerance (Thayale Purayil et al., 2020). Together with TFs, MAPK signaling cascades play important roles in ABA-dependent and ABA-independent plant abiotic stress responses (Mahmood et al., 2019). In conclusion, multiple metabolic pathways and signaling molecules are involved in plant defense against drought stress.

One of the major challenges facing plant science today is to discover mechanisms that enable plants to maintain productivity under adverse environmental conditions and to use this knowledge to produce crops that are better adapted to climate change (Li et al., 2021). Molecular mechanisms to drought stress have been extensively studied in broad-leaved species such as *Populus nigra* (Yildirim and Kaya, 2017), *Betula platyphylla* (Li H. Y. et al., 2020), and *Morus* spp. (Sun et al., 2020), but only a few studies have focused on *Haloxylon ammodendron* (Long et al., 2014; Fan et al., 2018) and *Haloxylon persicum* (Thayale Purayil et al., 2020). Therefore, it is important to understand the drought tolerance of *H. ammodendron* and *H. persicum*.

*H. ammodendron* and *H. persicum* are plants of the genus *Haloxylon* in the family Lyceae. They often form large-area pure stands in desert regions and have the function of fixing dunes. As xerophytic desert trees, these species have the characteristics of resistance to drought, high temperatures, saline alkali soils, wind erosion, and cold (Long et al., 2014; Thayale Purayil et al., 2020). The genus is widely distributed in desert and semi-desert areas, and the plants have ecological benefits. Therefore, they play an important role in maintaining the structure and function of the ecosystem, and thus revealing the drought-tolerance mechanism of *Haloxylon* is of great significance for drought-tolerant breeding. *Haloxylon* has a complex genetic background and a large genome that limit the study on the molecular mechanisms of drought tolerance (Lai et al., 2014). However, the development of next-generation sequencing (NGS, Illumina RNA sequencing) technology can overcome this difficulty (Thudi et al., 2012). Although the full-length transcript obtained by Illumina HiSeq has been completed, the length of the reads is insufficient, and this short-read sequencing hampers transcript reconstruction and annotation.

Single-molecule real-time sequencing (SMRT-seq) technology introduces powerful new tools (Rhoads and Au, 2015; Ardui et al., 2018). By virtue of its super long reading length advantage, SMRT-seq can directly obtain high-quality full-length transcript information without interruption and assembly (the average reading length of PacBio SMRT-seq is > 10 KB, and the actual length can reach 60 KB) (Van Dijk et al., 2018). However, according to current research, SMRT-seq often provides inaccurate gene information, and low gene coverage leads to a high error rate (Li et al., 2021). The incompleteness of Illumina HiSeq can be supplemented by more complete reading of SMRT-seq, while the inaccurate reading of SMRT-seq can be corrected by more accurate data of Illumina Hiseq (Li et al., 2021). Full-length transcripts that do not require assembly can be used to detect alternative splicing (AS) events, APA events, long non-coding RNAs (LncRNAs), and fusion transcripts. SMRT-seq has been successfully used to decipher the transcriptome of many plants, including *Larix kaempferi* (Li et al., 2021), *Crocus sativus* (Qian et al., 2019), *Dendranthema grandiflorum* (Zhao et al., 2018), and *C. obtusifolia* (Deng et al., 2018). Recently, a method combining SMRT-seq and Illumina HiSeq has been developed (Zhao et al., 2018; Zhang et al., 2019).

A previous study that screened candidate genes for *Haloxylon* drought stress mechanisms by the SMRT-seq technique obtained unclear results. In this study, SMRT and Illumina RNA-seq were used to study *H. ammodendron* (wet: soil water content 9.70–15.00%; drought: soil water content 2.41–4.00%) and *H. persicum* (wet: soil water content 3.38–5.12%; drought: Soil water content 1.05–3.11%) in both wet and dry ecological environments (Gong and Lü, 2017). First, the full-length transcriptome sequences from *H. ammodendron* and *H. persicum* obtained by Pacbio SMRT technology were described. These were then compared with Illumina RNA sequencing to generate a complete transcriptome. This study aimed to identify relevant genes and their molecular mechanisms in response to drought stress. In addition, this study also obtained the gene sequences of *H. ammodendron* and *H. persicum*, thereby providing a reference for further study of their transcriptomics.

## Materials and methods

### Sample plot setting

**FIGURE 1:**
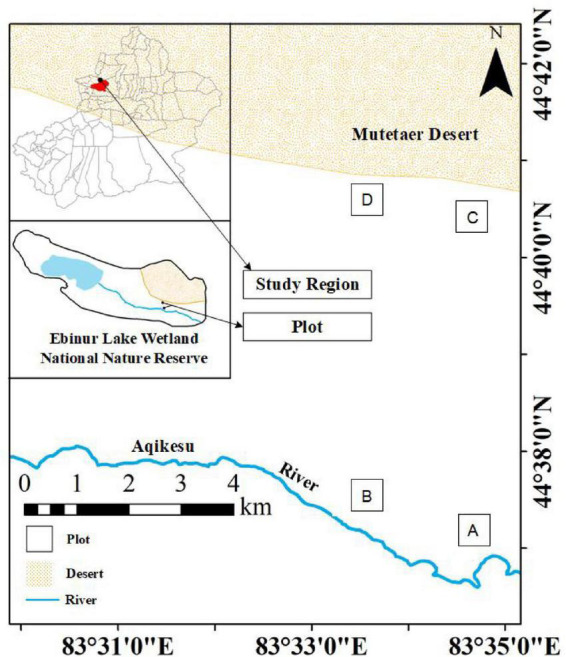
Study area and plot layout. The distribution area of *H. ammodendron*. **(A)** Humid and low salinity, and **(B)** arid and low salt; the distribution area of *H. persicum*, **(C)** humid and low salinity, and **(D)** arid and low-salinity.

Plants were sampled during the vigorous plant growth season from June to July 2021 at the field scientific observation station of the Ministry of Education of the temperate desert ecosystem in Jinghe County, Xinjiang University, starting from the East Bridge Management Station of Ebinur Lake Wetland Nature Reserve. Transects (width 0.1 km × 2.0 km) were set up perpendicular to the Aqiksu River in the areas with *H. ammodendron* and *H. persicum* and labeled as transect 1 (in the distribution area of *H. ammodendron*) and transect 2 (in the distribution area of *H. persicum*). According to the research results of previous studies (by this research group) (Gong and Lü, 2017), two soil water environments, humid and arid, were selected. A quadrat size of 50 m × 50 m was used in each of the two soil moisture environments [*H. ammodendron*: A: humid and low salinity (HS), and B: drought and low salinity (LS); *H. persicum*: C: humid and low salinity (HB), and D: arid and low salinity (LB)] (Figure 1). We investigated the number and abundance of woody plant species in the plots and selected five individuals of *H. ammodendron* and *H. persicum* with similar individual sizes regarding plant height, crown width, and base diameter for sampling and determination.

#### Determination of phenotypic and physiological indexes under drought stress

Use a meter ruler (accuracy: 0.1 cm) to measure the plant height, and use a vernier caliper (accuracy: 0.1 mm) to measure the base diameter, branch length and branch diameter.

Approximately 0.1 g of plant tissue (assimilative branches) was weighed, and 1 ml of acetone was added followed by homogenization in an ice bath. The sample was transferred to an EP tube, diluted to 1 ml with acetone, centrifuged at 4°C at 10,000 rpm for 10 min, and the supernatant was collected and kept on ice to be tested. Take the supernatant; put it on ice for testing; use enzyme labeling method to determine H_2_O_2_ content at a wavelength of 415 nm; and determine the catalase (CAT) activity at a wavelength of 240 nm (Satterfield and Bonnell, 1955; Johansson and Borg, 1988). Approximately 0.1 g of plant tissue (assimilative branches) was added to 1 ml of extract, ground into a homogenate using an automatic frozen ball mill, and then centrifuged at 12,000 rpm, 4°C for 10 min. Take the supernatant, put it on ice for testing, and measure the peroxidase (POD) activity by enzyme labeling method at a wavelength of 470 nm (Doerge et al., 1997). The data were averaged from three replicates.

#### Full-length cDNA library, RNA-seq, PacBio single-molecule real-time-seq and error correction

Total RNA isolation was done according to the protocol outlined by Jaakola et al. (2001), and total RNA was quantified and assessed using an Agilent Bioanalyzer 2100 (*Haloxylon ammodendron*: 25 s:18 s ≥ 1.6, RIN ≥ 6; *Haloxylon persicum*: 25 s:18 s ≥ 2.0, RIN ≥ 7). Total RNA from three biological replicates (*Haloxylon ammodendron*: HS1, HS2, HS3, LS1, LS2, LS3; *Haloxylon persicum*: HB1, HB2, HB3, LB1, LB2, LB3) was pooled in equal amounts, and 1 μg of the pooled RNA was used for cDNA synthesis and SMRT bell library construction. The purified RNA was reverse transcribed into cDNA using a SMARTer PCR cDNA Synthesis Kit (Clontech, Mountain View, CA, United States). The cDNA was amplified using a Kapa HiFi PCR kit (Kapa Biosystems, Wilmington, MA, United States). Size selection was carried out on a BluePippin (Sage Science, Beverly, MA, United States) system, and 1–2 kb, 2–3 kb, 3–6 kb, and 5–10 kb fractions were collected. After size selection, the collected cDNA fractions were treated with DNA damage repair mix followed by end repair and ligation of SMRT adapters using the PacBio SMRTbell Template Prep Kit (Pacific Biosciences, Menlo Park, CA, United States) to construct PacBio libraries. The library construction and sequencing were performed by Beijing Novogene Technology Co., Ltd. (China).

Illumina data were used to validate and quantify the PacBio-based transcripts. A total amount of 2 μg RNA per sample was used as an input material for the RNA sample preparations. RNA purity was checked using a kaiao K5500^®^ Spectrophotometer (Kaiao, Beijing, China). RNA integrity and concentration were assessed using the RNA Nano 6000 Assay Kit of the Bioanalyzer 2100 system (Agilent Technologies, Santa Clara, CA, United States). Sequencing libraries were generated using NEBNext^®^ Ultra™ RNA Library Prep Kit for Illumina^®^ (#E7530L, NEB, Ipswich, MA, United States) following the manufacturer’s recommendations. Each biological replicate was individually barcoded. Libraries were sequenced in 150-bp paired-end mode using an Illumina HiSeq X Ten.

The software SMRTlink v8.0 was used to filter and process the output. The parameters were as follows: minlength 50 (minimum length: 50 bp), MaxLength 15,000 (maximum length: 15,000 bp), and minpasses 1 (minimum number of full passes: The final data were considered as the valid data). The subreads in offline data subreads.bam file passed through the circular consensus sequencing (CCS) algorithm, i.e., self-correction of single-molecule multiple sequencing results to obtain a CCS. The CCS data were classified by detecting whether the CCS contained 5′-primer, 3′-primer, or poly-A and by identifying FLNC (full-length non-chimera) sequences and nFL (non-full length non-chimera) sequences. The FLNC sequences of the same transcript were clustered by hierarchical n * log (n) algorithm to obtain the consensus sequence. Finally, the full-length sequence was polished to obtain a consensus sequence for subsequent analysis. RNA-seq data were corrected by LoRDEC (Salmela and Rivals, 2014) software to further improve the sequencing accuracy, resulting in an accuracy of ≥ 99%.

#### Transcript de redundancy and gene function annotation

CD-HIT (Fu et al., 2012) software was used to cluster and compare protein or nucleic acid sequences through sequence alignment clustering and to remove redundant and similar sequences. We clustered the corrected transcripts according to the 95% similarity between sequences to remove redundancy.

To obtain comprehensive gene function information, gene function annotation was performed on the sequences after using the cd-hit software to remove redundancy. The databases used included NCBI non-redundant protein sequences (Nr), NCBI nucleotide sequences (Nt), Protein family (Pfam), and EuKaryotic Ortholog Groups/Clusters of Orthologous Groups of proteins (KOG/COG), a manually annotated and reviewed protein sequence database (Swissprot), the Gene Ontology (GO) database, and the Kyoto Encyclopedia of Genes and Genomes (KEGG) database (*E-value* < 1e^–5^).

#### CDS prediction, simple sequence repeat analysis, and prediction of long non-coding RNAs

We used ANGEL software for CDS prediction analysis (Shimizu et al., 2006). The program includes error-free and fault-tolerant modes (fault-tolerant mode is adopted by default). Genes were detected by SSR with MISA software. We used CNCI (Sun et al., 2013), PLEK (Li et al., 2014), and CPC2 (Kang et al., 2017) software and the Pfam (Finn et al., 2016) database to predict the coding potential of PacBio sequencing data, and subsequently analyzed the final LncRNAs.

#### Analysis of differentially expressed transcripts

To investigate the gene expression patterns of *H. ammodendron* and *H. Persicum* under drought stress (The experimental plots are located in arid areas, and are limited by water conditions all year round, resulting in lower soil water content the farther away from the river bank, and the long-term arid environment is affected by drought stress), we mapped all Illumina clean reads to the SMRT full-length transcriptome. The fragments per kilobase of exon per million fragments mapped (FPKM) values were then used to normalize the reads from the RNA-seq. To obtain the full-length transcripts that were differentially expressed between the comparison groups, the full-length transcripts were used as a reference; the second-generation data were used for quantitative comparison; and the differences between the groups were compared based on the quantitative read count. Using the model based on a negative binomial distribution, three biological repeats of DESeq2 were analyzed for differentially expressed genes (DEGs) between plants in the arid environment and plants in a humid environment. A *P*-value was assigned to each gene and adjusted by the Benjamini and Hochberg approach for controlling the false discovery rate. Genes with padj < 0.05 and | log_2_(FoldChange)| > 1 were identified as DEGs.

#### Gene Ontology and Kyoto Encyclopedia of Genes and Genomes enrichment analysis of differentially expressed transcripts

The Goseq R package was used for GO enrichment analysis of up-regulated and down-regulated DEGs between arid and humid samples (Young et al., 2010). We obtained the GO distribution of DEGs from three levels: Biological process (BP), molecular function (MF), and cell composition. Finally, KOBAS (3.0) software was used for KEGG pathway enrichment analysis to determine the pathways significantly enriched in DEGs (Wu et al., 2006).

#### Quantification and verification of gene expression levels

Ten DEGs were randomly selected for reverse transcription-quantitative PCR (qRT-PCR) assays to validate the reliability of the RNA-seq analysis. Reverse transcription was performed using a PrimeScript RT First Strand cDNA Synthesis Kit (Toyobo, Osaka, Japan). The first-strand cDNA synthesized from 1 μg of purified RNA was reverse transcribed using a Reverse Transcriptase kit (EP0442, Thermo Fisher Scientific). The qRT-PCR was carried out using an AceQ Universal SYBR qPCR Master Mix (Vazyme, Nanjing, China) system with PCR conditions following the manufacturer’s instructions. Quantitative RT-PCR reactions were conducted in 96-well plates with a real-time PCR System (Stepone plus, ABI) using the AceQ Universal SYBR qPCR Master Mix (Vazyme, Nanjing, China). Ha18SrRNA was used as an internal control. The 2^–ΔΔ*CT*^ method was used to determine the relative abundance of transcripts. For accuracy, the whole experiment was conducted thrice. All primers used in this study are listed in Supplementary Table 1.

#### Statistical analysis of data

All statistical analyses were performed using statistical package for the social sciences (SPSS) software version 20 (IBM, Chicago, IL, United States), and significant differences were evaluated by using analysis of variance. Different letters indicate significant differences at *P* < 0.05.

## Results

### Phenotypic and physiological changes of *Haloxylon ammodendron* and *Haloxylon persicum* under drought stress

**FIGURE 2:**
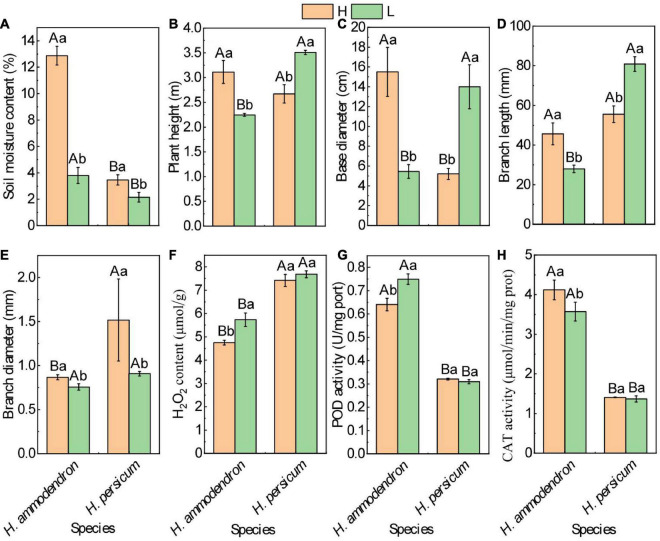
Effects of drought stress on phenotypic and biochemical parameters of *H. ammodendron* and *H. persicum*. **(A)** Soil moisture content; **(B)** plant height; **(C)** base diameter; **(D)** branch length; **(E)** branch diameter; **(F)** H_2_O_2_ content; **(G)** activities of POD; and **(H)** activities of CAT. H represents humid and low salinity.

*H. ammodendron* and *H. persicum* were used as experimental materials to study the effects of drought stress on growth and development as well as the regulatory mechanisms in response to drought stress. The growth of *H. ammodendron* and *H. persicum* was observed by measuring the difference in soil water content under wet and dry conditions. The soil water content of *H. ammodendron* decreased rapidly from 12.88 to 3.80%. The lower the soil water content, the greater the inhibition of growth by drought stress; this was manifested as clear decreases in plant height, the thinning of basal and branch diameters, and the shortening of branch length. The soil water content of *H. persicum* decreased from 3.46 to 2.16%, primarily through increases in plant height, basal diameter, and branch length and the thinning of branch diameter (Figures 2A–E). Physiological response measurements showed that the H_2_O_2_ contents of *H. ammodendron* (4.75–5.73 μmol/g) and *H. persicum* (7.42–7.68 μmol/g) accumulated under an arid environment (Figure 2F). Normally, the plants remove ROS through their own enzyme and non-enzyme antioxidant protection systems. The results showed that drought stress significantly decreased catalase (CAT) activity and increased POD activity (0.64–0.75 μmol/min/mg prot) in *H. ammodendron* (Figures 2G,H). *H. persicum* showed no significant changes.

### *Haloxylon ammodendron* and *Haloxylon persicum* transcriptome sequencing using PacBio single-molecule real-time-seq and RNA-seq

Short-read sequencing using the Illumina platform, the most widely used approach for RNA-seq, is a powerful technique for quantifying gene expression. The Pacific Biosciences SMRT-seq platform, which provides long reads up to transcript length, makes it possible to accurately reconstruct full-length splice variants. In total, 82 Gb of clean reads were produced by the Illumina HiSeq X Ten. The Q30 levels of *H. ammodendron* and *H. persicum* were greater than 93%, and the rate of clean reads was greater than 98% (Table 1). SMRT-seq showed that 20,246,576 and 8,908,053 subreads and 435,938 and 210,334 CCS reads were obtained from *H. ammodendron* and *H. persicum*, respectively. LoRDEC (Salmela and Rivals, 2014) software uses the second-generation data with high accuracy to correct the third-generation PacBio data. After multiple corrections, 35,096 and 20,621 consensus reads were obtained for *H. ammodendron* and *H. persicum* transcripts, respectively. According to the 95% similarity between sequences, the corrected transcripts were clustered to remove redundancy, and the numbers of transcripts remaining were 15,238 and 10,135 (Table 2).

**TABLE 1:** Illumina HiSeq of *H. ammodendron* and *H. persicum*, including three repetitions under humid (HS1, HS2, HS3 and HB1, HB2, HB3) and arid (LS1, LS2, LS3 and LB1, LB2, LB3) environments.

Sample	Raw reads (Mb)	Clean reads (Mb)	Clean bases(Gb)	Clean reads Q30 (%)	Clean reads rate (%)
HS1	43.08	42.37	6.35	93.29	98.35%
HS2	48.60	48.10	7.21	93.34	98.96%
HS3	48.96	48.32	7.25	93.1	98.69%
LS1	46.38	45.82	6.87	93.48	98.79%
LS2	48.06	47.37	7.11	93.52	98.57%
LS3	45.14	44.54	6.68	93.46	98.66%
HB1	47.10	46.45	6.97	93.54	98.63%
HB2	46.91	46.30	6.95	93.89	98.70%
HB3	48.05	47.44	7.12	93.07	98.72%
LB1	42.23	41.59	6.24	93.47	98.49%
LB2	41.78	41.19	6.18	93.06	98.59%
LB3	47.88	47.10	7.07	93.48	98.37%

LS1, LS2, LS3 and LB1, LB2, LB3, respectively, represent three repetitions of H. ammodendron and H. persicum in an arid environment; HS1, HS2, HS3 and HB1, HB2, HB3 denote three repetitions of H. ammodendron and H. persicum, respectively, under a humid environment.

**TABLE 2:** Overview of the single-molecule real-time sequencing in *H. ammodendron* and *H. persicum.*

Name		Subreads	Polymerases	CCS reads	Consensus reads	High-quality isoforms reads
Total number	SAB	20,246,576	491,508	435,938	35,096	15,238
	BAB	8,908,053	238,745	210,334	20,621	10,135
Mean length (bp)	SAB	1,286	56,104	1,868	1,860	1,982
	BAB	2,474	95,071	2,780	2,658	2,627

SAB and BAB are the names of mixed samples of H. ammodendron and H. persicum under two soil moisture conditions.

### Gene annotation and functional classification

**FIGURE 3:**
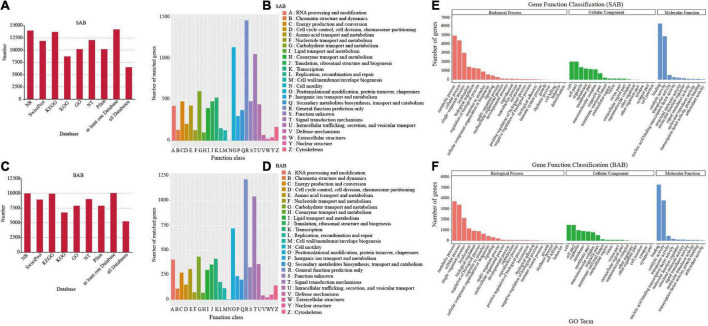
Gene annotation and functional classification. **(A)** Statistical chart of *H. ammodendron* transcripts annotated in 7 databases; **(B)** annotation results of *H. ammodendron* KOG distribution; **(C)** statistical chart of the results of annotation of the transcripts of *H. persicum* in 7 databases; **(D)** KOG distribution annotation results of *H. persicum*; **(E)** *H. ammodendron* GO annotation function diagram; **(F)** *H. persicum* GO annotation function diagram. SAB and BAB are the names of mixed samples of *H. ammodendron* and *H. persicum* under two soil moisture conditions.

To predict and analyze the functions of the unigenes, all full-length transcripts from SMRT were aligned to public databases: Nt, Nr, Pfam, SwissProt, KOG, GO, and KEGG (*E-value* < 1e^–5^). Respective totals of 14,187 and 10,037 transcripts of *H. ammodendron* and *H. persicum* were annotated using each database. The resulting values were 12,025 (84.76%) and 9,000 (89.67%) for Nt; 13,956 (98.37%) and 9,983 (99.46%) for Nr; 10,186 (71.80%) and 7,885 (78.56%) for Pfam; 11,843 (83.48%) and 8,912 (88.79%) for SwissProt; 8,692 (61.27%) and 6,721 (66.96%) for KOG; 10,186 (71.80%) and 7,885 (78.56%) for GO; and 13,667 (96.33%) and 9,902 (98.65%) for KEGG (Figures 3A,C). In addition, 6,555 and 5,292 transcripts of the two species were jointly annotated into five databases (Figures 3A,C). For unknown genes, the KOG (euKaryotic Ortholog Groups) method was used for functional annotation. For *H. ammodendron* and *H. persicum*, 9,736 and 7,570 genes, respectively, were annotated into the KOG database, and the number of KOGs under each category was counted (Figures 3B,D). There were 42,320 and 31,942 single genes annotated successfully in *H. ammodendron* and *H. persicum*, respectively. These were divided into 51 functional groups in three categories of GO: BP (19,259, 14,305), cellular component (CC) (10,453, 7,499), and MF (12,608, 10,138) (Figures 3E,F).

### Simple sequence repeat analysis and long non-coding RNA prediction

**FIGURE 4:**
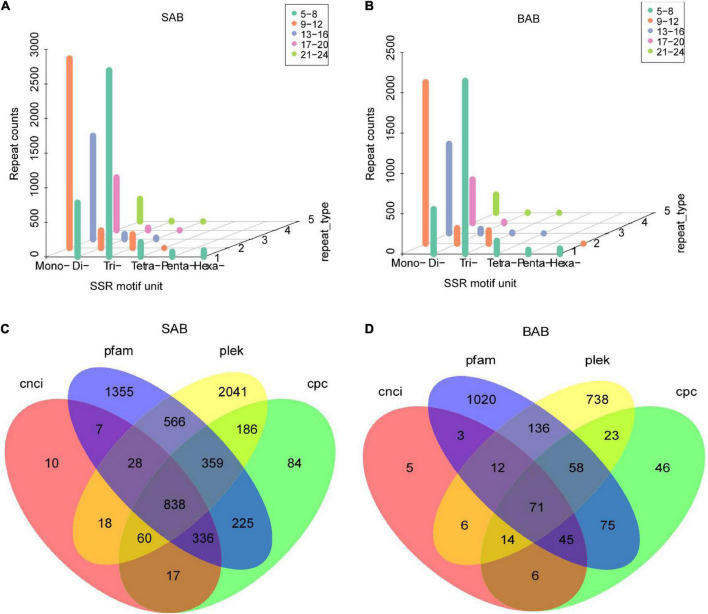
SSR analysis and forecasting for LncRNA. **(A)** SSR analysis of *H. ammodendron*; **(B)** SSR analysis of *H. persicum*; **(C)** Venn diagram of prediction results of LncRNA of *H. ammodendron*; and **(D)** Venn diagram of prediction results of LncRNA of *H. persicum*. SAB and BAB are the names of mixed samples of *H. ammodendron* and *H. persicum* under two soil moisture conditions; LS and LB, respectively, represent *H. ammodendron* and *H. persicum* in an arid environment; HS and HB denote *H. ammodendron* and *H. persicum*, respectively, under a humid environment.

SSRs are also known as short tandem repeats or microsatellite markers. SSR mining was carried out with MISA software. Totals of 9,794 and 7,330 SSR repeats were identified in *H. ammodendron* and *H. persicum* samples, respectively. From the whole samples of the two species, a large number of mono repeats were found followed by tri and di repeats (Figures 4A,B). LncRNAs (long-chain non-coding RNAs) are a type of RNA molecule with transcripts longer than 200 nt, and they do not encode proteins. Four different software (PLEK, CNCI, CPC, and Pfam) were used to predict the coding potential of SMRT-seq data to maximize accuracy. The numbers of predicted non-coding transcripts in *H. ammodendron* and *H. persicum* were: PLEK (4,096, 1,058), CNCI (1,314, 162), CPC (2,105, 338), and Pfam (3,714, 1,420) (Figures 4C,D). Venn diagrams were generated to show the specificity and complementarity of the results predicted by each software. The following figure visually shows the common and unique numbers of non-coding transcripts predicted by each method. To ensure the accuracy of prediction results, the common prediction results of each software were selected for subsequent analysis.

### Identification and analysis of differentially expressed genes

**FIGURE 5:**
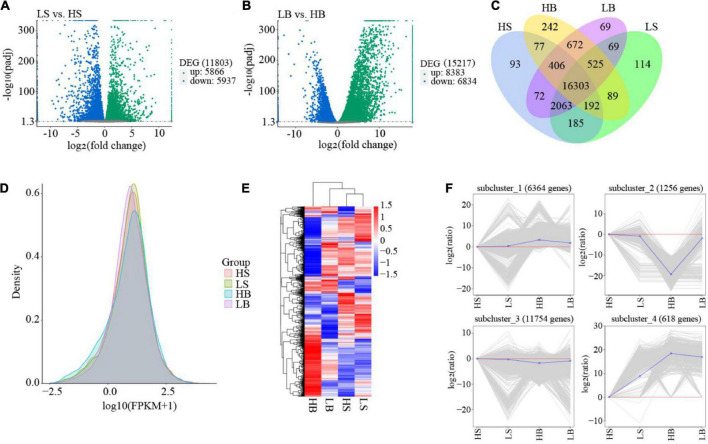
Overview of the RNA-seq data analysis. **(A)** The Volcano Plot of *H. ammodendron*. The *x*-axis represents the fold change of gene expression in different samples; the *y*-axis represents the statistical significance of the difference in gene expression; and the genes with significant differential expression are represented by green dots (up-regulated) and blue dots (down-regulated). **(B)** The Volcano Plot of *H. persicum.* The *x*-axis represents the fold change of gene expression in different samples; the *y*-axis represents the statistical significance of the difference in gene expression; and the genes with significant differential expression are represented by green dots (up-regulated) and blue dots (down-regulated). **(C)** Venn diagram of all samples of *H. ammodendron* and *H. persicum*; **(D)** FPKM density distribution; **(E)** DEG hierarchical clustering diagram of all samples; and **(F)** K-means clustering of gene expression trends. The gray line in each subgraph represents the relative expression of genes in a cluster under different experimental conditions (based on the expression of the first sample, i.e., shown by the red line), and the blue line represents the average relative expression of all genes in the cluster under different experimental conditions. LS and LB, respectively, represent *H. ammodendron* and *H. persicum* in an arid environment; HS and HB denote *H. ammodendron* and *H. persicum*, respectively, under a humid environment.

The FPKM density distribution map as a whole reflects the gene expression pattern of each sample. The graph shows a non-standard normal distribution. The area of each region is 1, and the sum of the representative probabilities is 1. The peak value of the density distribution curve represents the largest number of genes at this expression level. *H. ammodendron* and *H. persicum* had abundant gene expression, and drought stress affected the gene expression levels (Figure 5D). Using read counts as input, DESeq2 was used for DEG analysis, and the model based on a negative binomial distribution was used to compare plants in arid and humid environments. LS vs. HS and LB vs. HB had 11,803 (5,866 up-regulated and 5,937 down-regulated) and 15,217 (383 up-regulated and 6,834 down-regulated) DEGs [adjusted *P* < 0.05, | Log_2_(FoldChange)| > 1], respectively (Figures 5A,B). There were 16,303 DEGs between the two species (Figure 4C). A hierarchical cluster heat map was generated to view the expression patterns of the two species under different water conditions (Figure 5E). It can be seen from the figure that the gene expression of *H. ammodendron* and *H. persicum* could be divided into four groups, with significant differences among the groups. To further classify the expression patterns of all DEGs in each sample, K-means cluster analysis was performed (Figure 5F), and four subclasses with different average expression levels were identified. Cluster 2 and Cluster 4 contained genes that were expressed throughout the drought period. In addition, in the process of hierarchical clustering and K-means clustering, the expression levels of most genes showed significant changes between LS vs. HS and LB vs. HB, indicating that DEGs at the level of LS vs. HS and LB vs. HB transcripts could determine the drought-tolerance specificity of *H. ammodendron* and *H. persicum*.

**FIGURE 6:**
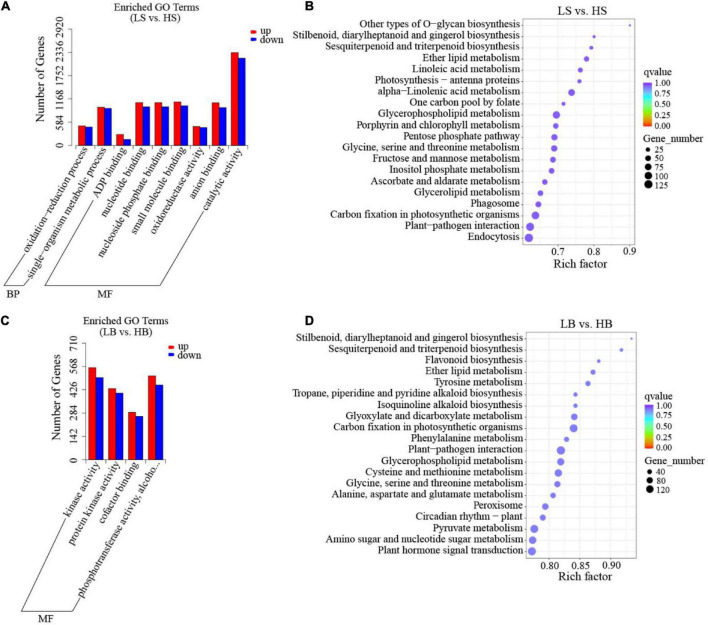
GO classification and KEGG enrichment analysis of DEGs. **(A)** GO classification of *H. ammodendron*; **(B)** KEGG enrichment analysis of *H. ammodendron*; **(C)** GO classification of *H. persicum*; and **(D)** KEGG enrichment analysis of *H. persicum*. LS and LB, respectively, represent *H. ammodendron* and *H. persicum* in an arid environment; HS and HB denote *H. ammodendron* and *H. persicum*, respectively, under a humid environment.

To determine the relative difference between DEGs and their products under drought stress, we performed the GO enrichment analysis for 46 functional groups of the BP category, the CC category, and the MF category. The first three GO terms of *H. ammodendron* were divided into two GO categories: “oxidation-reduction process” and “metabolic process” of the BP category; “binding,” “oxidoreductase activity” and “catalytic activity” for the MF category (Figure 6A and Supplementary Table 2). The first three GO terms of *H. persicum* were “kinase activity,” “cofactor binding,” and “phosphotransferase activity” of the MF category (Figure 6C and Supplementary Table 3). In the KEGG database, a total of 4,618 DEGs of *H. ammodendron* were enriched in 115 pathways, and 20 pathways were significantly enriched. The significant pathways included endocytosis, plant-pathogen interaction, carbon fixation in photosynthetic organisms, and glycerophospholipid metabolism (Figure 6B and Supplementary Table 2). For *H. persicum*, 5,916 DEGs were enriched in 115 pathways, of which 20 were significantly enriched, including carbon fixation in photosynthetic organisms, plant-pathogen interaction, glycerophospholipid metabolism, glyoxylate and dicarboxylate metabolism, peroxisome, pyruvate metabolism, flavonoid biosynthesis, plant hormone signal transduction, and amino sugar and nucleotide sugar metabolism (Figure 6D and Supplementary Table 3).

Glycerophospholipid metabolism, ether lipid metabolism, glycine, serine and threonine metabolism, sesquiterpenoid and triterpenoid biosynthesis, carbon fixation in photosynthetic organisms, plant-pathogen interaction and stilbenoid, diarylheptanoid and gingerol biosynthesis pathways were significantly enriched in both *H. ammodendron* and *H. persicum*, indicating that secondary metabolism and amino acid metabolism were the main metabolic mechanisms of *H. ammodendron* and *H. persicum* in response to drought stress.

### Differentially expressed genes related to drought stress signals in *Haloxylon ammodendron* and *Haloxylon persicum*

The perception and transmission of signals are crucial for plants under drought stress. A total of 319 (149 up-regulated and 170 down-regulated) and 415 (233 up-regulated and 182 down-regulated) DEGs involved in drought stress signal perception and transmission were found in *H. ammodendron* and *H. persicum*, respectively (Supplementary Table 4). Among these, 101 (55 up-regulated and 46 down-regulated) and 140 (76 up-regulated and 64 down-regulated) DEGs were involved in the synthesis and binding of ABA. For *H. ammodendron*, two genes encoding abscisic acid 8′- hydroxylase, one gene encoding abscisic acid receptor (PYL), two genes encoding 9-*cis*-epoxycarotenoid dioxygenase (NCED), 28 genes encoding protein phosphatase type-2C (PP2C), 16 genes encoding E3 ubiquitin-protein ligase (XERICO), and three genes encoding zeaxanthin epoxidase (ZEP) were significantly up-regulated under drought conditions. For *H. persicum*, one PYL (FC = 4.41), one NCED (FC = 41.44), four PP2C (FC = infinity), three SNF1-related protein kinase 2 (SnRK2) (FC = 2.03; FC = 7.84; FC = 6.80), three XERICO (FC = infinity), and one ZEP (FC = 20.37) genes were highly up-regulated. There were 182 and 237 DEGs in *H. ammodendron* and *H. persicum* that were identified as Ca^2+^ receptors, including calcium-transporting ATPase, calmodulin-binding protein (CaMBP), calcium-binding protein (CaBP), calcium-dependent protein kinase (CPK/CDPK), calmodulin (CAM), calmodulin-like protein (CML), calcineurin B-like protein (CBL), and CBL-interacting protein kinase (CIPK). In addition, most of the DEGs of MAPK and MAPKK components involved in the MAPK cascade in *H. ammodendron* and *H. persicum* were up-regulated. This indicated that the MAPK cascade played an important role in the response to drought stress.

### Transcription factors in response to drought stress in *Haloxylon ammodendron* and *Haloxylon persicum*

**FIGURE 7:**
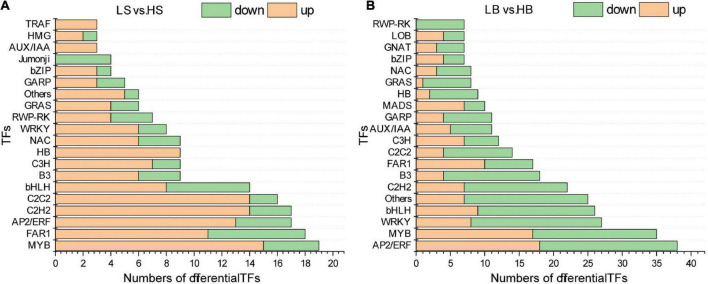
Differential transcription factors involved in drought stress (the top 20 differentially expressed were selected). **(A)** Different transcription factors of *H. ammodendron* involved in drought stress; **(B)** different transcription factors of *H. persicum* involved in drought stress. LS and LB, respectively, represent *H. ammodendron* and *H. persicum* in an arid environment; HS and HB denote *H. ammodendron* and *H. persicum*, respectively, under a humid environment.

Transcription factors are important regulatory genes in response to drought stress signals. In arid and humid environments, a total of 41 families of TFs of *H. ammodendron* were included in the DEGs (161 up-regulated and 56 down-regulated) (Supplementary Table 5). The largest group of TFs was the MYB followed by the FAR1, whereas other TFs belonged to the AP2/ERF, C2H2, C2C2, and basic helix-loop-helix (bHLH), and B3 families (*q* ≤ 0.05) (Figure 7A). For *H. persicum*, there were 398 (165 up-regulated and 233 down-regulated) differentially expressed TFs belonging to 50 families (*q* ≤ 0.05) (Figure 7B and Supplementary Table 5). The largest TF family was AP2/ERF followed by MYB, while other TFs belonged to WRKY, the bHLH family, others, and the C2H2 and B3 families. In addition to the common TFs, BBR-BPC (1), CSD (2), and HMG (3) were unique to *H. ammodendron*. TFs belonging to the ARID (1), CAMTA (1), CPP (1), mTERF (6), PHD (3), Rcd1-like (1), S1Fa-like (1), SWI/SNF-SWI3 (1), SWI/SNF-BAF60b (1), TAZ (1), Tify (2), and Whirly (1) families were unique to *H. persicum*. It is worth noting that FAR1 genes (Cluster-6558.3436, Cluster-6558.3407, Cluster-6558.1612, Cluster-363.0; Cluster-3181.2239, Cluster-3181.38499, Cluster-3181.261, Cluster-2360.1, Cluster-3181.43770, Cluster-629.0), AP2/ERF genes (Cluster-6558.1467, Cluster-6558.8862, Cluster-6558.23345; Cluster-3181.42435, Cluster-3181.42591, Cluster-3181.4445, Cluster-3181.12518; Cluster-3181.33858, Cluster-3181.4440, Cluster-3181.9216, Cluster-3181.18026, Cluster-3181.5615), MYB genes (Cluster-1167.0; Cluster-3181.1164, Cluster-3181.19228), C2H2 genes (Cluster-6558.3514; Cluster-1097.0, Cluster-11236.0), and bZIP genes (Cluster-1716.0; Cluster-3181.181, Cluster-3181.42470) of *H. ammodendron* and *H. persicum* were highly expressed under drought stress.

### Differentially expressed genes of *Haloxylon ammodendron* and *Haloxylon persicum* in response to drought stress

In this study, a total of 94 DEGs (46 up-regulated and 48 down-regulated) related to ROS scavenging were identified in *H. ammodendron* and 93 (56 up-regulated and 37 down-regulated) in *H. persicum.* These included superoxide dismutase (SOD), POD, catalase (CAT), glutathione peroxidase (GPX), ascorbic acid peroxidase (APX), glutathione S-transferase (GST), and glutathione reductase (GR) (Supplementary Table 6). Nine APX, four CAT, and seven SOD genes in *H. ammodendron* were up-regulated after drought stress, while 18 POD, 21 GST, and 3 GPX genes in *H. persicum* were significantly up-regulated.

Lignin is a component of plant cell walls, and the content and composition of lignin may be altered under drought stress. As shown in Supplementary Table 5, 79 (49 up-regulated and 30 down-regulated) and 103 (50 up-regulated and 53 down-regulated) DEGs in *H. ammodendron* and *H. persicum* were related to lignin biosynthesis. These DEGs included those encoding 4-coumarate-CoA ligase (4CL), Beta-glucosidase, caffeoyl-CoA *O*-methyltransferase (CCOMT), caffeic acid 3-*O*-methyltransferase (COMT), cinnamoyl-CoA reductase (CCR), cinnamyl alcohol dehydrogenase (CADH), Laccase, Caffeoylshikimate esterase (CSE), ferulate-5-hydroxylase(F5H) and phenylalanine ammonia-lyase (PAL) (Supplementary Table 7).

In addition, other types of DEGs may be related to the drought stress responses of *H. ammodendron* and *H. persicum* (Supplementary Table 8). Totals of 80 (42 up-regulated and 38 down-regulated) and 114 (73 up-regulated and 41 down-regulated) DEGs were identified in *H. ammodendron* and *H. persicum*, belonging to heat shock protein (HSP), late embryogenesis-abundant (LEA) protein, abscisic stress-ripening (ASR) and aquaporin (AQP). It is noteworthy that most of these DEGs were highly expressed under drought stress, for example, Hsp90 (transcript_HQ_SAB_transcript8389/f2p0/2478, FC = Infinity) gene in *H. ammodendron* and HSP90 (transcript_HQ_SAB_transcript29992/f3p0/870, FC = 136.84) gene in *H. persicum*.

Sugar metabolism in plants becomes complex due to changes under drought stress (Du et al., 2020). In this study, 349 DEGs (197 up-regulated and 150 down-regulated) in *H. ammodendron* and 435 (250 up-regulated and 185 down-regulated) in *H. persicum*, were identified as being related to glucose metabolism (Supplementary Table 9). These DEGs were involved in starch/sucrose, fructose/mannose, amino sugar/nucleoside sugar, pentose/glucuronate, galactose, pentose phosphate, and glycolysis/gluconeogenesis metabolism.

### Quantification and verification of gene expression level

**FIGURE 8:**
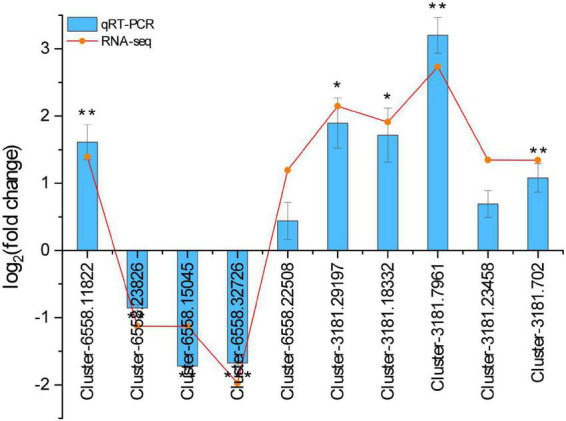
The relative expression levels of 10 DEGs between RNA-seq and qRT-PCR. The gene relative expression levels were determined by the 2^– ΔΔ*CT*^ method. RNA-seq, RNA sequencing; qRT-PCR, quantitative real-time polymerase chain reaction. All statistical analyses of data were performed using SPSS software (version 26, IBM, Chicago, IL, United States) to assess significant differences (*n* = 3). Significance was defined as *(*P* < 0.05), **(*P* < 0.01), and ***(*P* < 0.001).

To verify the reliability of RNA-seq data of *H. ammodendron* and *H. persicum* under drought stress, 10 DEGs were randomly selected for qRT-PCR. There was a strong similarity between the qRT-PCR results and RNA-seq data (Figure 8), indicating that the RNA-seq data were reliable.

## Discussion

### Characteristics of PacBio single-molecule real-time-seq and analysis of long non-coding RNA

*H. ammodendron* and *H. persicum* are extremely important wind- and sand-fixing plants, and as such, they are known as the guardians of the desert. However, they have been affected by drought stress for a long time. It is important to understand the drought-tolerance mechanism of these tree species. In this study, samples of *H. ammodendron* and *H. persicum* were collected under two soil environments (wet and dry), and SMRT-seq and Illumina Hiseq were used to reveal the defensive responses of *H. ammodendron* and *H. persicum* to drought stress.

SMRT-seq was able to obtain long reading or full-length transcripts, and more Illumina Hiseq single genes were obtained by mapping to SMRT-seq. The transcriptomes of *H. ammodendron* and *H. persicum* were sequenced using the Pacific Biosciences SMRT-seq platform; 35,096 and 20,621 unigenes were successfully collected, with 9,404 and 8,426 unigenes of 2,000–3,000 bp, and 4,281 and 6,603 unigenes of more than 3,000 bp. Therefore, a large number of candidate genes were detected by combining Illumina Hiseq and SMRT-seq. LncRNAs can be used as competitive endogenous RNA (ceRNA) to participate in the plant response to drought stress, representing a new gene regulatory layer (Pang et al., 2019; Ramirez et al., 2021). In this study, 838 and 71 LncRNAs were predicted in *H. ammodendron* and *H. persicum* samples, respectively. However, their functions need further study. Therefore, future study of the functions of these LncRNAs will help to better understand the response of *H. ammodendron* and *H. persicum* to drought stress at the molecular level.

### Protein kinases related to Ca^2+^, mitogen-activated protein kinase, and abscisic acid signaling pathways under drought stress

The regulation of drought tolerance in plants is a complex trait involving multiple genes. Under drought stress, the induction of signal transduction pathway-related genes is a common phenomenon, for example, the induction of the Ca^2+^ signal pathway, the MAPK signal pathway, the ABA signal pathway, and downstream regulatory pathways related to physiological adaptation (Mahmood et al., 2019; Lamers et al., 2020). Among the signal pathways of drought stress, the ABA signal pathway is important in signal transduction (Li et al., 2021). ZEP is an enzyme for zeaxanthin epoxidation in the ABA biosynthesis pathway. Overexpression of *AtZEP* enhances the tolerance of plants to drought stress (Park et al., 2008). NCED is a rate-limiting enzyme in the ABA biosynthesis pathway, and the *AtNCED3* gene is highly induced under drought stress (Sato et al., 2018). Under drought stress, *H. ammodendron* had three ZEP genes, and *H. persicum* had one *NCED3* homologous gene up-regulated. This result is consistent with the up-regulation of the NCED homologous genes of *Gossypium* spp. and *Vitis riparia* under drought stress, indicating that the ABA signaling pathway plays an important role in the response of plants to drought stress (Khadka et al., 2019; Mahmood et al., 2019). When the intracellular ABA level increases, the ABA receptor PYR/PYL/RCA protein binds to PP2C and releases SnRK2s (Kalapos et al., 2016). After that, SnRK2s can phosphorylate downstream substrates to actively activate the ABA response (Mahmood et al., 2019). SnRK2s protein is the main protein kinase that can transmit ABA signals throughout the entire plant (Zhang et al., 2022). In this study, one *PYL9* and one *PYL8*, 29 and 41 PP2C, and one and four SnRK2 genes of *H. ammodendron* and *H. persicum* were up-regulated, indicating that they are active in the ABA signal regulation system and play an important role in the process of drought tolerance.

As a secondary messenger, Ca^2+^ plays an important role in plant drought stress. Plant Ca^2+^ sensor proteins include calmodulin (CaMs), CBL, and Ca^2+^-dependent protein kinases (CDPKs) (Aldon et al., 2018). CaM can target a variety of enzymes and structural proteins, while CBL only specifically targets a group of CDPKs designated as CIPK (Luan, 2002; Cui et al., 2018). Under drought stress, 70 and 98 CaM genes and 10 and 9 CIPK genes were significantly changed in *H. ammodendron* and *H. persicum*, respectively. These results support the view that the Ca^2+^ signaling pathway plays an important role in *H. ammodendron* and *H. persicum* under drought stress.

The MAPK signal is considered as a secondary signal regulation mechanism in response to drought stress (Zhu, 2016). The MAPK cascade signal pathway is composed of three types of protein kinases, MAP kinase kinase kinase (MAPKKK), MAP kinase kinase (MAPKK), and MAPK. MAPK amplifies and transmits external stimulus signals through MAPKKK-MAPKK-MAPK stepwise phosphorylation and activates stress response gene expression (Bigeard and Hirt, 2018). It was found that overexpression of the maize *ZmMAPK1* gene could enhance the drought resistance of transgenic *Arabidopsis* plants (Wu et al., 2015). Weng et al. (2014) also found that overexpression of *BnMAPK1* in rape could enhance its drought resistance by increasing the water retention of cells and the activity of roots. In this study, the transcriptional levels of DEG encoding MAPK (12 and 9 genes), MAPKK (4 and 4 genes), and MAPKKK (2 and 6 genes) were up-regulated in *H. ammodendron* and *H. persicum*. KEGG pathway enrichment analysis showed that DEGs of *H. ammodendron* and *H. persicum* enriched the MAPK signal pathway. The data indicated that the MAPK signaling pathway is an important part of the drought stress response mechanism.

### Transcription factor-related genes are key components of the drought response mechanism of *Haloxylon ammodendron* and *Haloxylon persicum*

Drought stress response is controlled by complex regulatory networks in plants. TFs are important regulators in this network, and they play key roles by activating or repressing the expression of downstream stress-related target genes (Xuan et al., 2022). For example, *AtFAR1* can strongly respond to drought stress, while *FAR1* can positively regulate the ABA signaling pathway and integrate light and ABA signaling to better adapt plants to environmental stress (Tang et al., 2013). Persak and Pitzschke (2014) found that *MYB44-REP* overexpression reduced drought stress tolerance in *Arabidopsis*. According to previous studies, the *AtMYB2* protein is involved in ABA-induced gene expression under drought stress (Yao et al., 2022). In *Arabidopsis*, drought, salinity, salicylic acid (SA), and H_2_O_2_ treatments up-regulated *AtWRKY46* expression (Ding et al., 2014). In *Gossypium hirsutum*, the *GhWRKY41* (Chu et al., 2016) gene regulates the production of ROS in the organism in an ABA-dependent manner (Ong et al., 2020), and in transgenic *Nicotiana benthamiana*, the homologous gene positively responds to drought and salt stress. Most studies have shown that multiple WRKYs respond to different abiotic stresses (especially drought and salt stress), for example, *TaWRKY1*, *TaWRKY33* (He et al., 2016), and *TaWRKY46* (Li X. R. et al., 2020). In addition, genes of the NAC, bZIP, and bHLH families are also involved in many drought stress responses (Li et al., 2021; Xuan et al., 2022). In this study, *H. ammodendron* identified 217 DEGs belonging to 41 TF families, including one WRKY DEG (Cluster-6558.4158), one Maf DEG (Cluster-1716.0), three ERF DEGs (Cluster-6558.23345, Cluster-6558.8862, and Cluster-6558.1467), three MYB DEGs (Cluster-7507.0, Cluster-13816.0, and Cluster-6558.8062), and four FAR1 DEGs (Cluster-6558.3436, Cluster-6558.3407, Cluster-6558.1612, and Cluster-363.0) that were markedly increased. *H. persicum* had four WRKY DEGs, one WRKY (Cluster-3181.31636) DEG, three bZIP DEGs, and six MYB DEGs, including two MYB (Cluster-11703.0, Cluster-3181.1164) DEGs, six FAR1 (Cluster-3181.261, Cluster-2360.1, Cluster-3181.2239, Cluster-3181.38499, Cluster-3181.43700, Cluster-629.0) DEGs that were highly expressed under drought stress. Given that TFs belonging to different families are expressed under drought induction, it can be inferred that TFs play a central role in the complex drought resistance regulatory network of *H. ammodendron* and *H. persicum*.

### Antioxidative mechanisms against drought stress

Drought stress can lead to excessive accumulation of ROS in plants, inevitably causing oxidative damage (Tang et al., 2022). As the first enzyme in antioxidant action, SOD removes O^2–^ and produces H_2_O_2_ (Aleem et al., 2022). POD is an important regulator of H_2_O_2_ balance in plants, as it scavenges H_2_O_2_ to produce H_2_O (Wang et al., 2009). GST and CAT protect plants from ROS damage (Sarker and Oba, 2018; Wahibah et al., 2018). Therefore, SOD, CAT, and GST are important antioxidant enzymes in plants. Under drought stress, SOD, POD, and GST genes were significantly differentially expressed in *H. ammodendron* and *H. persicum* (Supplementary Table 5). *H. persicum* accumulated more H_2_O_2_ than *H. ammodendron*, but H_2_O_2_ increased more than in *H. persicum*. The POD activity of *H. ammodendron* showed a significant upward trend, while the CAT activity showed a significant downward trend. There were no significant changes in the POD or CAT activities of *H. persicum* (Figures 2E–H).

### The roles of functional proteins, lignin biosynthesis, and sugar metabolism in the responses of *Haloxylon ammodendron* and *Haloxylon persicum* to drought stress

During drought stress, the expression levels of many genes were significantly up-regulated in *H. ammodendron* and *H. persicum*, for example, AQPs, HSP, and ASR (Supplementary Table 5). AQPs are a class of channel proteins responsible for the transport of water molecules. They are primarily involved in passive transport processes, and they participate in regulating the water balance of the entire plant under drought conditions (Song et al., 2022). The resistance of tomato PIP2;7 and PIP2;5 overexpression lines to drought or osmotic stress was significantly enhanced (Wang et al., 2014). There were 14 (12 up-regulated and 2 down-regulated) and 20 (6 up-regulated and 14 down-regulated) DEGs involved in AQPs regulation in *H. ammodendron* and *H. persicum*, including 1 PIP1-3 (FC = 1.52), 2 PIP1-4 (FC = 2.41; FC = 2.01), 2 PIP1 (FC = 2.31; FC = 1.96), and 1 PIP2-7 (FC = 1.73) genes; PIP1 (FC = Infinity), PIP1-4 (FC = 271.92), and PIP2-1 (FC = 7.23) genes in *H. persicum*. Both were significantly up-regulated. The ASR family is part of the LEA protein family, and it can improve plant drought tolerance through an ABA-mediated pathway (Liang et al., 2019). The regulation of ASR in the two species was opposite; most of the genes encoding ASR in *H. ammodendron* were down-regulated, while in *H. persicum* these were mostly up-regulated. As molecular chaperones, HSP proteins play a role in the cellular stability of living plants from environmental stressors such as drought, high temperature, and salinity (Park and Seo, 2015). HSP90, one of the most abundant proteins in eukaryotes, typically targets small but critical signaling proteins, folds them when denatured under stress, and releases them after other signaling molecules bind to these proteins (Yamada and Nishimura, 2008). Overexpression of *AtHSP90* may alter the normal homeostasis of calbindin and disrupt the normal Ca^2+^ signaling pathway, thereby making transgenic plants overexpressing HSP90 more tolerant to high calcium concentrations and leading to increased tolerance to salt and drought stress (Song et al., 2009). The genes encoding HSP90 (FC = Infinity) and HSP90-5 (FC = Infinity) in *H. ammodendron*, and two HSP90-2 (FC = 18.06; FC = Infinity), HSP90-5 (FC = 41.88) and HSP90-6 (FC = 16.52) genes in *H. persicum* were highly expressed under drought stress. Therefore, functional proteins play an important role in the mechanism of plant drought resistance.

As a major component of secondary cell walls, lignin plays an important role in plant resistance to drought stress (Cao et al., 2020; Gu et al., 2020). Recently, it has been shown that the increase of lignin enhances the tolerance of *P. ussuriensis* to drought stress (Li et al., 2022). Monomer lignin proceeds through the styrene-acrylic biosynthetic pathway (Liu et al., 2020). In this study, the expression levels of genes involved in lignin biosynthesis of *H. ammodendron* (such as 4CL, Beta-glucosidase, CCR, and COMT) and those involved in lignin biosynthesis of *H. persicum* (such as Beta-glucosidase, CADH, COMT, and Laccase) were increased. Similar results were obtained in *Paeonia ostii* (Zhao et al., 2021) and *L. kaempferi* (Li et al., 2021), suggesting that plants may enhance tolerance to drought stress by promoting lignin biosynthesis acceptability.

Under drought stress, sugar allocation, metabolism, and transport in plants are significantly altered (Du et al., 2020). Studies have shown that under drought conditions, non-structural carbohydrates such as sucrose and sugar alcohols accumulate in cells and increase osmotic stress tolerance (Tomasella et al., 2019). Zanella et al. (2016) showed that β-amylase catalyzes the breakdown of starch into soluble sugars, and overexpression of *AtADH1* in *Arabidopsis* improved total soluble sugar accumulation and enhanced resistance to abiotic stress (Shi et al., 2017). In this study, eight β-amylase DEGs of *H. ammodendron* and seven ADH1 DEGs of *H. persicum* were up-regulated, possibly indicating that *H. ammodendron* and *H. persicum* increase soluble sugars to adapt to drought stress.

### Schematic model of *Haloxylon ammodendron* and *Haloxylon persicum* response to drought stress

**FIGURE 9:**
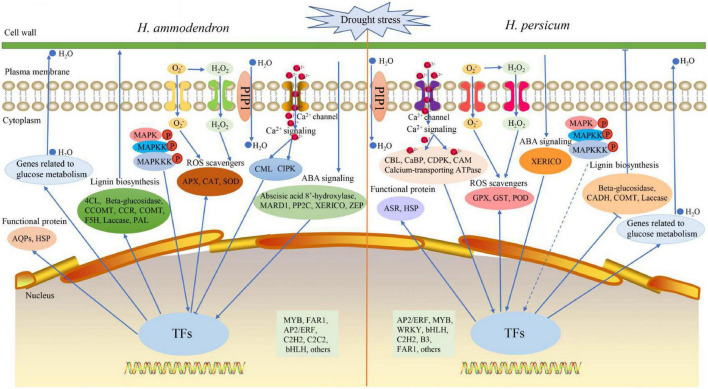
A hypothetical model of drought tolerance mechanism for *H. ammodendron* and *H. persicum*. Arrows indicate positive regulation, lines ending in bars indicate negative regulation, dashed arrows indicate neither up regulation nor down regulation.

Based on the main contents of drought-responsive DEGs and their associated pathways/networks, we proposed a schematic model (Figure 9). Under drought stress, the expression of a large number of genes in *H. ammodendron* and *H. persicum* changed, including stress signal sensing genes, anti-oxidative stress genes, functional protein genes, lignin biosynthesis genes and sugar metabolism genes. When threatened by drought stress: (1) plant cells can sense stress signals and transmit them from extracellular to intracellular through the regulation of ABA (abscisic acid 8′-hydroxylase, PYL, NCED, PP2C, ZEP, SnRK2, XERICO), Ca^2+^ (CaMBP, CBL, calcium-transporting ATPase, CaM, CML, CIPK, CDPK, CaBP), and MAPK (MAPK, MAPKK, MAPKKK) signaling cascade-related protein kinase genes that are then transcribed by related TFs (MYB, FAR1, AP2/ERF) in the nucleus that regulate the expression of related genes. (2) A large amount of ROS is produced in cells under drought stress, and this may cause oxidative damage to cells (Zhang et al., 2022). The ROS scavenging system can neutralize excess ROS (Wan et al., 2022). TFs promote the up-regulation of ROS scavenging system-related genes (APX, CAT, SOD; GPX, GST, POD) and reduce the ROS content in cells to reduce their damage to cells. (3) To reduce the damage of drought stress, the osmotic regulation-related genes in *H. ammodendron* and *H. persicum* were regulated by TFs in the nucleus. The up-regulation of β-amylase and ADH1 increases the synthesis of soluble sugars, and macromolecular proteins such as (AQP and HSP; ASR and HSP) begin to accumulate in large quantities, which can balance the intracellular and extracellular water potential to a certain extent. In addition, PIP proteins located on the cell membrane and PIP proteins located on the vacuolar membrane can also regulate the water balance in cells and vacuoles. (4) Drought stress can lead to ROS accumulation and other changes in the cell wall (Zhu, 2016). Lignin is the main component of secondary cell walls, and drought stress promotes the increased expression of lignin biosynthesis genes (4CL, Beta-glucosidase, CCOMT, CCR, COMT, F5H, Laccase; Beta-glucosidase, CADH, COMT, Laccase), thereby enhancing plant drought tolerance.

## Conclusion

The responses of *H. ammodendron* and *H. persicum* to drought stress were studied through the synergy of physiological and molecular responses. Compared with *H. ammodendron*, the growth environment of *H. persicum* is harsher (lower soil water content), resulting in greater growth and physiological changes of *H. ammodendron* in response to drought, and the response was faster and more intense. The Illumina RNA-seq and SMRT long-read sequences were integrated to obtain the full-length transcripts of *H. ammodendron* and *H. persicum*. In addition, LncRNAs and SSRs were obtained, and DEGs involved in drought regulation were analyzed. We found that the Ca^2+^ signaling pathway, the MAPK cascade, and the ABA signaling pathway play important roles under drought conditions, but the genes involved in their regulation vary greatly in number and expression patterns. The study also found that TFs such as FAR1, AP2/ERF, MYB, and WRKY were involved in the response to drought stress, but most of the genes involved in regulating of *H. ammodendron* were up-regulated, on the contrary, *H. persicum*. Physiological index measurements and bioinformatic observations showed that the expression patterns of genes related to ROS balance, glucose metabolism, functional proteins, and lignin biosynthesis were different between the two species under drought stress, indicating that *H. ammodendron* and *H. persicum* have different regulatory mechanisms and drought tolerance in response to drought stress. This study is the first step to establishing the full-length unparameterized transcriptome of *H. ammodendron* and *H. persicum*, and the results provide a foundation for further research on their regulatory mechanisms.

## Data availability statement

The original contributions presented in this study are publicly available. This data can be found here: NCBI, PRJNA855227 available at: https://submit.ncbi.nlm.nih.gov/subs/.

## Author contributions

FY and GL conceived the study. FY collected the data, analyzed the data, and drafted the text. Both authors edited the manuscript.
