# Supplementary material for: Characterization of the gene expression profile response to drought stress in Haloxylon using PacBio single-molecule real-time and Illumina sequencing
Source: Front Plant Sci. 2022 Aug 16;13:981029. doi: 10.3389/fpls.2022.981029 (PMC9424927; doi:10.3389/fpls.2022.981029)
Supplement: Supplementary file 1 [file Table_1.DOCX]

**Table S1 Sequences of primers used in qRT-PCR.**

| **Gene ID** | **Primer sequences** | **T_m_** | **Length(bp)** |
| --- | --- | --- | --- |
| Cluster-6558.11822-F | ACTTTTTGGGACGGAAGGAGT | 59.51 | 79 |
| Cluster-6558.11822-R | ACGTAGTGGAAGGGGGAGTT | 60.18 |  |
| Cluster-6558.23826-F | GGGCGCACCTTGGAATATAATG | 59.77 | 109 |
| Cluster-6558.23826-R | CATTGTTGGGGAAGCATCTTGG | 60.09 |  |
| Cluster-6558.15045-F | CTCGGCTACCATGTGAGAAGAC | 60.48 | 178 |
| Cluster-6558.15045-R | ATCCACTACTTGAGCCACCAG | 59.44 |  |
| Cluster-6558.32726-F | GGAGACTGATGCAACACCCA | 59.96 | 190 |
| Cluster-6558.32726-R | GTCCTTGCTCCTTCTGACCA | 59.31 |  |
| Cluster-6558.22508-F | GTTCCATTTACCTGTGTGGCTT | 59.11 | 125 |
| Cluster-6558.22508-R | CAATAGTCACTCTGAAGAACGGC | 59.39 |  |
| Cluster-3181.29197-F | TGTGCTGTTTGGATGCCTGA | 60.18 | 197 |
| Cluster-3181.29197-R | TCCATGCCATCACTACTCGC | 59.9 |  |
| Cluster-3181.18332-F | GTACAGCCGCTCAATCCTCT | 59.54 | 111 |
| Cluster-3181.18332-R | CATGTGCATCAATAGGGTGGG | 59.04 |  |
| Cluster-3181.7961-F | TCATCATCAAAGCTGCCGGA | 59.75 | 240 |
| Cluster-3181.7961-R | CGAACAAGGAATGCCCGTGAA | 61.48 |  |
| Cluster-3181.23458-F | TGGACATTTAGGCGAACCACA | 59.93 | 98 |
| Cluster-3181.23458-R | AGTCTTCCTGTCGCGCTTATC | 60.2 |  |
| Cluster-3181.702-F | CTCGGCAATGGAGCCACTTA | 60.11 | 165 |
| Cluster-3181.702-R | ATTTGCCAAAGCAGCCCAAG | 59.96 |  |
| Ha18SrRNA-F | CTCTGCCCGTTGCTCTGATGAT |  |  |
| Ha18SrRNA-R | CCTTGGATGTGGTAGCCGTTTC |  |  |

**F: Forward primer (5'→3'); R: Reverse primer (5'→3').**
